# From Awake to Minimalist Spontaneous Ventilation Thoracoscopic Lung Surgery: An Ongoing Journey

**DOI:** 10.3390/jcm14072475

**Published:** 2025-04-04

**Authors:** Eugenio Pompeo

**Affiliations:** 1 Unit of Thoracic Surgery, Department of Surgical Sciences, University of Rome Tor Vergata, 00133 Rome, Italy; pompeo@uniroma2.it; Tel.: +39-0620902877; 2 Division of Thoracic Surgery, University Hospital Policlinico Tor Vergata, 00133 Rome, Italy

**Keywords:** awake thoracic surgery, spontaneous ventilation thoracic surgery, nonintubated thoracic surgery, video-assisted thoracic surgery, regional anesthesia

## Abstract

Spontaneous ventilation lung surgery (SVLS) without intubation is aimed at avoiding adverse effects of mechanical ventilation lung surgery (MVLS) entailing one-lung mechanical ventilation through a double-lumen tracheal tube. This innovative strategy has evolved following the publication of a small randomized study of thoracoscopic pulmonary wedge resection carried out under spontaneous ventilation without tracheal intubation in fully awake patients. It now entails target-controlled sedation, the use of a laryngeal mask, and thoracic analgesia by intercostal or paravertebral blocks and has shown promise both in unicenter and multicenter studies, resulting in optimal feasibility and safety and highly satisfactory results, particularly in patients undergoing lung cancer resection and metastasectomy, lung biopsy for undetermined interstitial lung disease, lung volume reduction surgery for end-stage emphysema, and bullectomy for primary and secondary spontaneous pneumothorax. However, concerns and unresolved issues still exist regarding the advantages and disadvantages of SVLS as well as the identification of optimal indications. This perspective is aimed at providing a critical overview of the current knowledge about SVLS with emphasis on recent data comparing the results with those of MVLS published in the last 10 years.

## 1. Introduction

The modern history of spontaneous ventilation lung surgery (SVLS) without intubation was marked in 2004 by the publication of a small randomized study which reported the feasibility and outcomes of video-assisted thoracic surgery (VATS) pulmonary wedge resection carried out under thoracic epidural analgesia with spontaneous ventilation without tracheal intubation in fully awake patients [1]. At that time, this anecdotally mentioned, novel approach [2,3] raised several concerns, including whether adequate oxygenation could be maintained in a spontaneously ventilating patient following the creation of a surgical pneumothorax, and how well patients would tolerate being fully conscious during the surgical procedure.

It is worth noting, however, that in the early experiences, the main adverse effects associated with SVLS were not related to maintaining satisfactory perioperative oxygenation, which was successfully managed even in patients with severe respiratory impairment [4]. Instead, the primary issues regarded the development of permissive hypercapnia due to spontaneous ventilation within an open chest environment, and patient discomfort from remaining conscious throughout the procedure [5]. Interestingly at that time, patients who more easily accepted awake SVLS were often older and those with significant respiratory compromise, who may have perceived general anesthesia with intubation and mechanical ventilation as riskier and preferred the challenge of staying awake during surgery.

Since then, protocols for modern SVLS have evolved to incorporate target-controlled sedation to maintain spontaneous ventilation while ensuring unconsciousness. Non-invasive ventilation assistance to assure airway patency and provide additional oxygen can be accomplished by a laryngeal mask, whereas thoracic analgesia is now preferentially achieved through intercostal or paravertebral blocks. Overall, these changes have improved patient safety and comfort, allowing comprehensive physiological monitoring [6].

Initial reports have shown that the early fears regarding the intolerability of a surgical open pneumothorax during spontaneous ventilation [7] were proven to be scientifically unfounded [1,2,3,4,5].

But why was SVLS considered advantageous compared to the established mechanical ventilation lung surgery (MVLS) entailing one-lung ventilation through a double-lumen tracheal tube?

In fact, while the first attempts at SVLS date back to the beginning of the 20th century [7,8], it was not until the introduction of one-lung ventilation via double-lumen tube intubation [9], which rapidly became the standard anesthesia in thoracic surgery, that the physiopathology of MVLS and its potential adverse effects became well understood [10].

In our experience, the most compelling evidence supporting SVLS has emerged from the early results of an original lung volume reduction surgery (LVRS) method carried out through an awake SVLS anesthesia protocol that we had developed [4,11] in an attempt to reduce the significant mortality and morbidity historically reported in patients with severe emphysema operated on through MVLS anesthesia [12,13]. We had noticed that in these instances, the most critical postoperative period began immediately after surgery during weaning, when adverse effects of tracheal intubation, deep anesthesia, and mechanical ventilation could jeopardize the rapid restoration of valid, spontaneous ventilation. As a result, prolonged intubation or even re-intubation was reported to be necessary in up to 11.5% of cases [14], despite the adoption of VATS [15] being less invasive than the initially proposed median sternotomy [16]. On the other hand, in our experience, avoiding tracheal intubation and mechanical-ventilation-related adverse effects abolished the need for intensive care unit stays in patients undergoing LVRS by SVLS, and facilitated a quicker recovery of respiratory function after surgery [4].

Other patient subgroups who have been shown to benefit from this approach include those with interstitial lung disease (ILD) requiring lung biopsy for diagnostic purposes, geriatric patients undergoing lung cancer resection [17], and young and healthy patients requiring simple surgical procedures, such as management of spontaneous pneumothorax [3].

Experienced surgical teams dedicated to SVLS have consistently reported that anatomical lung resections, including lobectomies and segmentectomies, can be performed safely using SVLS, and notably, even more complex surgical procedures, such as sleeve lobectomy [18] and tracheal surgery [19], have been performed using this modality. This leads to the question of whether the feasibility of performing a complex procedure by SVLS indicates that it is equally safe compared to the same procedure carried out by MVLS.

This perspective is aimed at providing a critical overview of the current knowledge about SVLS with emphasis on recent data comparing the results with those of MVLS published in the last 10 years, and on the basis of previous findings and our own experience on this topic.

## 2. Technical Details

### 2.1. Anesthesia

Several technical aspects of SVLS anesthesia have evolved over time. Initial protocols in fully awake patients [1] have undergone significant changes, particularly with the adoption of target-controlled sedation, with or without the use of non-invasive ventilation supraglottic devices such as laryngeal masks [20]. The advantages of using a laryngeal mask include being able to maintain of a patent airway; provide additional oxygen, along with the possibility to temporarily assist ventilation by low-positive-pressure ventilation whenever required; monitor end-tidal carbon dioxide tension; and correct permissive hypercapnia. This device can prove particularly useful in patients with respiratory impairment as well as in obese subjects.

Permissive hypercapnia can develop during SVLS due to hypoventilation induced both by a surgical pneumothorax and pendular ventilation [21]. Nonetheless, hypercapnia rarely becomes clinically dangerous and may even exert potentially beneficial effects, including increased lung compliance and improved ventilation/perfusion matching [22].

Additionally, the original choice of analgesia via thoracic epidural catheterization has increasingly been replaced by simpler methods including intercostal, paravertebral, and other thoracic muscle blocks, performed either through transcutaneous puncture [23] or from inside the pleural cavity under thoracoscopic vision [24]. This shift can be attributed to an attempt to avoid potential adverse effects associated with epidural analgesia, such as headache, urinary retention, hypotension, and even more severe complications like spinal cord injury [25] and epidural hematomas [26]. In addition, the multilevel block induced by thoracic epidural analgesia can prove unnecessary whenever single-access thoracoscopic approaches are preferred.

Regarding sedation, most of the current literature on SVLS reports the use of target-controlled sedation with precise monitoring through tools like the bispectral index [6,20] or entropy [27]. More frequently, sedation is achieved by titrated infusion of propofol, sufentanil, and/or remifentanil. Recently, intravenous infusion of dexmedetomidine has also been reported in order to reduce opioid consumption [28].

In order to limit coughing reflexes stimulated by pulmonary hilum manipulation, intrathoracic block of the vagus nerve and/or aerosolized local anesthetics on the lung surface are commonly employed, particularly when performing anatomical lung resection [6]. Moreover, temporary unilateral block of the phrenic nerve has also been attempted in order to reduce intraoperative diaphragmatic contractions and lung movements during SVLS [29].

Due to the lack of an endotracheal tube and of positive-pressure mechanical ventilation in a subject ventilating spontaneously, the achievement of complete lung re-expansion at end-procedure may prove more difficult or incomplete because of the surgically induced pneumothorax in the non-dependent lung. However, with the development of adjuvant transthoracic negative-pressure ventilation methods, full lung re-expansion can easily be achieved during SVLS without the need for any non-physiological positive-pressure ventilation [30]. In this respect, in one study from our group, negative-pressure ventilation resulted in better oxygenation measures than the application of positive-pressure ventilation through a laryngeal mask.

Whenever a rapid conversion to MVLS is needed due to uncontrolled hypoxemia/hypercapnia, excessive diaphragmatic and lung movements, the presence of dense and diffuse pleural adhesions, or the need to perform an emergency thoracotomy, intubation with a double-lumen tube in lateral decubitus can prove challenging and requires specific expertise of the anesthesia team.

Finally, intraoperative discomfort, sometimes leading to panic attacks, has not been reported since target-controlled sedation protocols were introduced.

### 2.2. Surgical Technique

As far as surgical technique details are concerned, these as well have evolved over time. In fact, the concept itself of SVLS, which implies the aim of an overall minimized invasiveness of the surgical procedure, has fitted nicely with other technical innovations developed to reduce thoracic pain while improving the cosmetic results, such as uniportal access [31], the reduced adoption of any kind of tube and catheter including chest tubes [32], leading to the so-called tubeless thoracic surgery, and the adoption of improved surgical technologies, including advanced cutting and coagulation devices such as novel thin-tip harmonic scalpels (Harmonic 1100, Johnson & Johnson medical Spa, Pratica di Mare, Italy) and motorized endoscopic staplers (Echelon 3000, Johnson & Johnson medical Spa, Pratica di Mare, Italy), which, when summed up with all the aforementioned choices and tools, have lead us to define minimalist thoracic surgery as one aimed at optimizing surgical outcomes while minimizing the procedure-related invasiveness [33].

Amongst the technical concerns that can be raised against SVLS, one of the main concerns entails the potential risk associated with performing delicate surgical maneuvers, such as isolating the pulmonary hilum vessels in spontaneously ventilating patients, without secure endotracheal control of the airway. In these instances, maintenance of physiological diaphragmatic contractions, leading to rhythmic lung movements, can complicate surgical manipulation, particularly in cases of intraoperative bleeding that require rapid and precise surgical intervention.

## 3. Outcome Measures

Initial investigations on SVLS focused primarily on feasibility and safety, using standardized thoracic surgery outcome measures. These included subjective assessments, such as the surgeon’s evaluation of technical feasibility and patients’ satisfaction scores [1], the need for conversion to MVLS, operative mortality, morbidity, and length of hospital stay [1,2,3,4,5]. However, since, in most instances, surgical procedures performed via SVLS or MVLS are technically identical, with the only distinction being anesthesia protocols, to strengthen the potential advantages of SVLS, the selection of appropriate outcome measures is crucial. Moreover, although it might be expected that standard outcome measures, such as morbidity and hospital stay, may differ significantly in certain high-risk subgroups even within small sample sizes, it has been reported that in simple procedures performed on relatively healthy, young patients, such as bullectomy for spontaneous pneumothorax, SVLS shows non-inferiority compared to MVLS rather than superiority [20]. This supports the idea that MVLS anesthesia protocols, including double-lumen tube intubation, curarization, and single-lung mechanical ventilation, though currently considered the standard anesthesia protocols required for lung surgery, may represent unnecessary anesthesia over-management for simple lung surgery procedures [34].

Once the overall feasibility and safety of SVLS were established, the potential advantages of this strategy were investigated across different surgeries. To assess differences between the two anesthesia protocols, respiratory function parameters, such as the ratio between oxygen tension and the fraction of inspired oxygen (PaO_2_/FiO_2_), to be assessed early after surgery, and at different time points, have already provided some evidence of better results in a selected cohort undergoing SVLS [4]. On the other hand, carbon dioxide arterial tension (PaCO_2_) has been shown to increase more during SVLS, though returning toward baseline values more rapidly than following MVLS [4,35].

Another outcome measure used to compare SVLS versus MVLS is the total time spent in the operating room, summing up operative, anesthesia, and recovery room times, which has been shown to be reduced following SVLS [1,5]. This metric is aimed at not only providing insights about technical aspects but also acting as a surrogate for the overall postoperative physiological patient status, as assessed by the anesthesiologist prior to discharge from the recovery room.

Different studies have compared the costs of SVLS versus MVLS. In particular, anesthesia-related costs resulted in being lower with SVLS in the management of spontaneous pneumothorax [36,37] and resection of non-small cell lung cancer (NSCLC) [38], and the overall costs proved to be lower in SVLS lung metastasectomy [39].

Other outcome measures that have been evaluated include postoperative fasting time [40], which has also shown better results after SVLS, suggesting quicker recovery times towards normality.

Amongst the other potential advantages of SVLS, there is a lower postoperative increase in white blood cells [41,42] and inflammatory mediators such as interleukin-6 [7], better preserved natural killer lymphocytes [43,44], and lower cortisol levels [45] early after surgery, as also reported in a systematic review with a meta-analysis [46]. Additionally, recent findings suggest other physiological derangements, including better cerebral oxygenation perioperatively [47,48] and better postoperative preservation of cognitive function, as indicated by higher neurocognitive test scores in an SVLS group at 24 h and 6 months as compared to an MVLS group [6].

## 4. Indications

### 4.1. Lung Cancer Resection

Despite the initial technical concerns regarding the routine adoption of SVLS for the surgical treatment of NSCLC, this indication is one of the most extensively investigated in the recent literature. One reason for this may be that NSCLC resection is the primary indication for thoracic surgery and includes the most common surgical procedures performed by thoracic surgeons globally. Nonetheless, the geographical distribution of studies on this topic underlines that, so far, the adoption of SVLS is not uniformly applied throughout the world but is rather preferentially adopted in certain countries, particularly in some Asian countries, probably reflecting differences in dedicated research programs and health management policies. In fact, out of 13 papers published in the last decade, 6 were from China, 3 from Taiwan, and 1 from Thailand, Saudi Arabia, Hungary, and USA. Of these, 11 were retrospective analyses, 9 of which included propensity score matching, and only 2 were prospective randomized studies. It is worth mentioning that eight studies did not specify a primary outcome, and one included multiple primary outcomes, while one study assessed cognitive function scores. Additionally, two studies included both disease-free survival and overall survival as primary outcomes, one of which showed better results in the SVLS group. Morbidity was reported in 10 studies, with 2 showing a significant advantage for SVLS, while hospital stay was reported in 9 studies, 6 of which showed a significant reduction in hospital stay for SVLS patients (Table 1).

### 4.2. Lung Volume Reduction Surgery

Emphysema is among the most debilitating chronic lung diseases, often leading to respiratory failure, necessitating long-term oxygen therapy, and eventually resulting in frequent exacerbations and high risks of mortality. When pharmacologic treatment, respiratory rehabilitation, and oxygen therapy fail to assure an acceptable quality of life, surgical options such as LVRS and lung transplantation can be considered. Unfortunately, despite being associated with a significant clinical benefit, LVRS by MVLS anesthesia protocols has shown 90-day mortality between 4.6 and 5.9% and major pulmonary morbidity, defined as the need for reintubation, tracheostomy, ventilator support for more than 2 days, or pneumonia within 30 days, affecting nearly 30% of patients in the large NETT trial [13,56]. These features prompted the search for less invasive interventional methods, including endobronchial valve placement via flexible bronchoscopy [57]. In 2006, we described an original LVRS method carried out by SVLS in awake patients under thoracic epidural anesthesia [11], which proved safe and feasible, becoming our surgical treatment of choice in patients with end-stage emphysema. Notably, in a unicenter randomized study, we showed that LVRS by SVLS resulted in improved early postoperative outcomes, including better oxygenation measures within one hour post-surgery, no operative mortality, no need for intensive care unit stay, lower morbidity, and shorter hospital stay, compared to LVRS performed by MVLS [4]. In addition, in the same study, improvements in forced expiratory volume in one second, residual volume, six-minute walking test, dyspnea index, and physical function quality of life scores paralleled the results of LVRS under MVLS [4].

During the last decade, our surgical method has been refined to further minimize invasiveness and now entails target-controlled sedation, thoracic analgesia by intercostal block with injection of bupivacaine and lidocaine carried out immediately before a single surgical incision, and the use of novel 60 mm motorized endoscopic staplers and a small-sized single chest tube, to realize an ultra-mini-invasive surgical procedure denominated minimalist quasilobar LVRS. This surgical option allowed for further reducing postoperative morbidity and resulted in a greater reduction in residual volume compared to two previously adopted LVRS methods [33].

Recently, satisfactory results of LVRS by SVLS have also been reported in patients with severe hypercapnia by another center in Germany [58].

### 4.3. Lung Biopsy for ILD

Lung biopsy for undetermined ILD is another optimal indication for SVLS, particularly as these patients have been shown to be high-risk for even simple wedge resections if carried out using intubation and MVLS protocols, particularly in patients with low lung diffusion capacity and recent exacerbations [59]. In this setting, VATS biopsy by SVLS has been shown to be simple and highly reliable, making it easily reproducible [60].

In the last 10 years, several single-center studies [60,61,62,63], one multicenter retrospective analysis [64], and a recent meta-analysis [65] have investigated this indication. Specifically, a review with a meta-analysis by Patirelis et al. [65] demonstrated that, out of 675 patients across 13 studies, operative mortality was zero, and morbidity ranged from 0.0% to 27.3%, with a median of 7.0%. The most common complications were prolonged air leaks (23.4%), pneumonia (7.8%), and atelectasis (6.2%). Hospitalization ranged from a mean of 1 day to a median of 8 days. Diagnostic yield ranged from 84.6% to 100%, with a mean of 97.1%. The meta-analysis comparing SVLS and MVLS approaches demonstrated a median morbidity rate of 5.2% for SVLS versus 24% for MVLS, with a significantly lower complication risk in the former group (odds ratio, 0.31; CI, 0.16–0.59). Diagnostic yield was comparable between the two groups, approaching 100% in both. In comparison with alternative interventional diagnostic methods, SVLS biopsy appears less invasive than bronchoscopic methods like cryobiopsy, offering lower mortality and morbidity risks and superior diagnostic yields due to the collection of large, architecturally intact lung tissue specimens. A recent study from Cherchi et al. [66] reported that even in obese patients with ILD, lung biopsy by SVLS, though associated with longer operative time than in the control group, did not result in a higher morbidity rate and achieved a diagnostic yield of 99%.

The geographical distribution of studies published from 2015 to 2024 includes eight centers in Europe, four in Asia, three in America, and one in Africa [65].

### 4.4. Bullectomy

Bullectomy for primary spontaneous pneumothorax is considered one of the optimal indications for SVLS since these patients are usually young with a slim body habitus and a normal respiratory function, and the surgical procedure is technically quick and simple. For all these reasons, the expected outcome is not to demonstrate superiority over MVLS, but rather to offer simpler anesthesia management with nearly equivalent clinical results. Following the anecdotal report from Nezu et al. in 1997 [3], in a small randomized study published in 2007 [35], we showed that the global operating room time, visual analogue pain scores (VAS), nursing care calls, hospital stay, and overall costs were lower in the awake SVLS group compared to the MVLS control group. More recently, of six comparative articles published in the last 10 years, two were randomized studies (one multicenter) [21,67], and five were retrospective analyses, including both single-center [36,37,68,69] and multicenter studies [70].

In one randomized study by Liu et al. [20] comparing 335 patients undergoing bullectomy either by SVLS or MVLS, intraoperative and postoperative morbidity did not differ significantly between the groups, leading the authors to conclude that SVLS bullectomy was non-inferior to MVLS in terms of complication rates. In another small randomized study by Hwang et al. [67], pain VAS at 1 h post-surgery was significantly better in the SVLS group. Similarly, in the multicenter retrospective study by Elkhouly et al. [70], morbidity was equivalent between the SVLS and MVLS groups, but hospital stay was significantly shorter in the SVLS group. Similar results have been reported in another retrospective study by Palaniappa et al. [68]. Recurrence rates, assessed in two studies, showed no intergroup differences (Table 2).

The geographical distribution of comparative studies, aside from the multicenter analysis, includes two studies each from China and Korea and one each from Malaysia and Turkey.

In addition to surgical management of primary spontaneous pneumothorax, bullaplasty for giant bullous emphysema and surgical management of secondary pneumothorax [71,72] by SVLS have also been reported, with promising unicenter results. One of the criticisms addressed to SVLS bullectomy is that, using this strategy, lung reinflation at end-procedure to test the presence of an air leak may not be as effective as when using MVLS due to the lack of tracheal intubation.

### 4.5. Other Indications

Other indications for SVLS include lung metastasectomy, first reported in 2007 [5]. In 2017, Ambrogi et al. [39] updated their unicenter experience, comparing the results of 48 patients with those of a control group of 13 patients who underwent MVLS metastasectomy. The mean number of lesions resected per patient was 1.51, and intergroup comparisons showed a shorter hospital stay and lower costs in the SVLS group, with equivalent intergroup morbidity and long-term outcomes.

Another noteworthy investigational indication for SVLS reported within the last 10 years relates to a series by Akopov et al. [73] involving 42 patients who underwent VATS abscessoscopy and drainage of acute infectious pulmonary destruction under local anesthesia and sedation. In this series, there was one operative death and satisfactory overall results.

## 5. Future Perspectives

Published data about SVLS within the last 10 years suggest superiority or non-inferiority compared to MVLS with an increasing level of scientific evidence regarding selected outcomes and indications (Table 3).

There exist several areas that may merit attention as far as future investigation into SVLS is concerned. One key area is a better assessment of the potential physiological advantages of this strategy compared to MVLS. Additional knowledge will also help to clarify which patients may benefit most from MVLS, with particular emphasis on patients with advanced age, poor pulmonary function, ILD, and neoplastic diseases requiring lung surgery, as well as patients with associated comorbidity. In this respect, the use of SVLS for lung biopsy of undetermined ILD is being increasingly adopted thanks to the highly satisfactory results, with no operative mortality, negligible morbidity, and optimal diagnostic rates.

Hopefully, further exploration may be devoted to SVLS in patients with end-stage emphysema, in whom LVRS by SVLS has been shown to offer significant advantages in the early postoperative period and equivalent intermediate-term benefits to MVLS. Despite representing a niche indication, LVRS by SVLS may be considered, particularly by centers with multidisciplinary experience in the treatment of severe emphysema and those with a lung transplantation program.

The adoption of MVLS for simple lung procedures, such as lung wedge resection, metastasectomy, and particularly bullectomy for spontaneous pneumothorax, constitutes indications for which there already exists some robust evidence of easier and faster anesthesia management with non-inferior results compared to MVLS.

The reliability of using SVLS for anatomic lung resections in NSCLC treatment should still be considered investigational, though promising results have already been reported in dedicated centers, particularly in Asia. Still, the undeniable advantage of performing an anatomical lung resection in an immobile surgical field, as in MVLS, versus performing the same maneuvers in a rhythmically moving surgical field, as during SVLS, cannot be understated considering that the latter requires additional care and expertise due to the technical challenges involved. Thus, an accurate assessment of the risk-to-benefit ratio may help in deciding which patients can really take advantage of a technically more challenging SVLS procedure.

Another intriguing area of investigation concerns the potential beneficial effects of SVLS on immunologic function early after surgery, which might play a protective role against tumor spread and recurrence and eventually improve the survival of patients with primary and metastatic lung tumors.

Finally, SVLS warrants more thorough testing within other mini-invasive options including robot-assisted and minimalist surgical strategies, which, thanks to continuing technological advancements, may contribute to rendering properly selected thoracic surgery procedures safer, less invasive, and highly effective.

It is likely that additional data resulting from large prospective studies will eventually help to demonstrate not only which surgical procedures may be performed by SVLS but particularly which are really worth performing, either because of superior outcomes or equivalent results, including simpler anesthesia management.

## Figures and Tables

**Table 1:** Studies comparing SVLS versus MVLS resection of non-small cell lung cancer published between 2015 and 2024.

First Author	Year	Country	Study Design	Patients SVLS/ MVLS	Anatomic/ Wedge	Primary Outcome	Primary Outcome Results	Morbidity (%)	Hospital Stay (Days)
Udelsman, B.V. [23]	2024	USA	Retrospective with PSM	30/60	28/2 vs. NI	NI	-	6.0 vs. 15.0	2.0 vs. 2.0
Yu, J. [49]	2024	Thailand	Retrospective	54/78	Lobectomy	NI	-	3.7 vs. 11.5	4.0 vs. 5.0
Wang, L. [48]	2024	China	Randomized non-inferiority	60/60	48/12 vs. 51/9	NI	-	1.7 vs. 1.3	4.1 vs. 4.5
Hsiung, P.Y. [6]	2024	Taiwan	Randomized	54/53	16/38 vs. 21/32	NFS	69.9 vs. 65.3	6.1 vs. 9.0	3.0 vs. 3.0
Farkas, A. [50]	2023	Hungary	Retrospective with PSM	32/35	Lobectomy single-port	NI	-	3.1 vs. 25.7	-
Wang, C. [17]	2022	China	Retrospective with PSM	80/80 Age > 65 y	58/22 vs. 61/19	NI	-	17.7 vs. 17.2	7.5 vs. 13.8
Chen, P.H. [51]	2022	Taiwan	Retrospective with PSM	79/158 Age > 75 y	29/50 vs. 88/70	OS + DFS	*p* = 0.99 *p* = 0.56	7.6 vs. 12.0	-
Yang, F. [41]	2022	China	Retrospective with PSM	73/73	20/53 vs. 23/50 single-port	NI	-	2.7 vs. 9.6	4.0 vs. 4.5
Wang, R. [52]	2022	China	Retrospective with PSM	31/62 PPF	31/0 vs. 62/0	NI	-	16.0 vs. 24.1	7.7 vs. 10.0
Zheng, J. [53]	2021	China	Retrospective with PSM	194/200	Lobectomy	5-y% OS + DFS	90.8 vs. 82.7 * 85.0 vs. 76.3 *	-	-
Wang, M.L. [54]	2021	Taiwan	Retrospective with PSM	97/97	Lobectomy	5-y% OS + DFS	98 vs. 94 86 vs. 74	-	-
Al Ghamdi, Z.M. [55]	2018	Saudi Arabia	Retrospective	30/30	Lobectomy	NI	-	20.0 vs. 20.0	6.9 vs. 7.6
Liu, J. [40]	2016	China	Retrospective with PSM	136/136	Lobectomy (116) Segmentectomy (20)	Fasting time, (+ others)	6.7 vs. 12,3 h 6.5 vs. 13.8 h	8.6 vs. 10.3 15.0 vs. 15.0	7.4 vs. 8.6 6.0 vs. 8.3

SVLS: spontaneous ventilation lung surgery; MVLS: mechanical ventilation lung surgery; PSM: propensity score matching; NI: not indicated. OS: overall survival; DFS: disease-free survival; * *p* < 0.05.

**Table 2:** Studies comparing SVLS versus MVLS bullectomy for primary spontaneous pneumothorax published between 2015 and 2024.

First Author	Year	Country	Study Design	Patients SVLS vs. MVLS	Surgical Procedure	Primary Outcome	Morbidity	Hospital Stay (Days)	Recurrence (%)
Palaniappa, P.M. [68]	2023	Malaysia	Retrospective	54/36	Bullectomy	Hospital stay	2 vs. 3	3.0 vs. 4.5 *	-
Yanik, F. [69]	2023	Turkey	Retrospective with PSM	60/60	Bullectomy	-	12% vs. 13%	-	5.1 vs. 6.4
Elkhouly, A.G. [70]	2022	Egypt	Multicenter retrospective with PSM	70/70	Bullectomy/pleurodesis	Morbidity + recurrrence	8.0% vs. 9.0%	3.5 vs. 5.8 *	6 vs. 8 at 24 months
Liu, J. [20]	2022	China	Multicenter randomized non-inferiority	162/163	Bullectomy	Morbidity (intraop + postop)	17.9% vs. 22.09%	Log rank *p* = 0.49	-
Jung, J. [37]	2019	Korea	Retrospective with PSM	52/52	Bullectomy	-	5 vs. 4 (air leaks only)	5.4 vs. 9.2 *	1.9 vs. 5.8
Hwang, J. [67]	2018	Korea	Randomized	21/20	Bullectomy	Pain VAS 1.43 vs. 4.35 * (postop 1 h)	-	-	-
Guo, Z. [36]	2016	China	Retrospective	15/22	Bilateral bullectomy	-	0 vs. 2	4.5 vs. 5.2	7.0 vs. 5.0

MVLS: mechanical ventilation lung surgery; PSM: propensity score matching; SVLS: spontaneous ventilation lung surgery; * *p* < 0.05.

**Table 3:** Outcomes of SVLS versus MVLS according to indications and types of published data in the period 2015–2024.

Outcome Measure	Bullectomy for Primary Spontaneous Pneumothorax		Lung Resection for Undetermined ILD		LVRS for End-Stage Emphysema		Lung Resection for Cancer	
	Better	Non-Inferior	Better	Non-Inferior	Better	Non-Inferior	Better	Non-Inferior
Postoperative PaO_2_/FiO_2_	-	3	2	-	2	-	-	-
Postoperative PaCO_2_ (mmHg)	-	3	-	-	-	-	-	-
Thoracic pain (VAS)	2	3	2	-	-	-	2	-
Operative time (min)	2	3	2	-	2	-	2	-
Anesthesia time (min)	2	3	2	-	2	-	-	-
Global operating room time (min)	2	-	2	-	2	-	2	-
Intraoperative cerebral oxygenation	-	-	-	-	-	-	2	-
Postoperative cognitive function	2	-	-	-	-	-	2	-
Operative mortality rate	-	-	-	-	-	-	-	-
Operative morbidity rate	2	3	2	-	2	-	2	2
Hospital stay	2	3	2	-	2	-	2	-
Stress and inflammatory response	3	-	-	-	-	-	2	-
Cellular immune function	-	-	-	-	-	-	2	-
Procedure-related costs	2	3	-	-	2	-	2	-
Disease-free survival	-	-	-	-	-	-	2	-
Overall survival	-	-	-	-	-	-	2	-

Legend: 0: no adequate scientific data; 1: data shown in retrospective analysis only; 2: data shown in unicenter randomized studies or in retrospective studies with propensity score matching; 3: data shown in multicenter randomized studies. ILD: interstitial lung disease; MVLS: intubated mechanical ventilation lung surgery; LVRS: lung volume reduction surgery; SVLS: non-intubated spontaneous ventilation lung surgery; VAS: visual analogue scale.
